# Src-Mediated Cross-Talk between Farnesoid X and Epidermal Growth Factor Receptors Inhibits Human Intestinal Cell Proliferation and Tumorigenesis

**DOI:** 10.1371/journal.pone.0048461

**Published:** 2012-10-31

**Authors:** Zhongsheng Peng, Jean-Pierre Raufman, Guofeng Xie

**Affiliations:** Division of Gastroenterology and Hepatology, VA Maryland Health Care System, University of Maryland School of Medicine, Baltimore, Maryland, United States of America; II Università di Napoli, Italy

## Abstract

Besides its essential role in controlling bile acid and lipid metabolism, the farnesoid X receptor (FXR) protects against intestinal tumorigenesis by promoting apoptosis and inhibiting cell proliferation. However, the mechanisms underlying these anti-proliferative actions of FXR remain to be elucidated. In the present study, we examined the effects of FXR activation (FXR overexpression and treatment with an FXR agonist GW4064) and inactivation (treatment with FXR siRNA and an FXR antagonist guggulsterone) on colon cancer cell proliferation *in vitro* using human colon cancer cell lines (H508, SNU-C4 and HT-29) and *in vivo* using xenografts in nude mice. Blocking FXR activity with guggulsterone stimulated time- and dose-dependent EGFR (Tyr845) phosphorylation and ERK activation. In contrast, FXR overexpression and activation with GW4064 attenuated cell proliferation by down-regulating EGFR (Tyr845) phosphorylation and ERK activation. Treatment with guggulsterone and GW4064 also caused dose-dependent changes in Src (Tyr416) phosphorylation. In stably-transfected human colon cancer cells, overexpression of FXR reduced EGFR, ERK, Src phosphorylation and cell proliferation, and in nude mice attenuated the growth of human colon cancer xenografts (64% reduction in tumor volume; 47% reduction in tumor weight; both P<0.01). Moreover, guggulsterone-induced EGFR and ERK phosphorylation and cell proliferation were abolished by inhibiting activation of Src, EGFR and MEK. Collectively these data support the novel conclusion that in human colon cancer cells Src-mediated cross-talk between FXR and EGFR modulates ERK phosphorylation, thereby regulating intestinal cell proliferation and tumorigenesis.

## Introduction

The farnesoid X receptor (FXR, NR1H4), a member of the nuclear receptor superfamily of ligand-activated transcription factors, is highly expressed in the liver and gastrointestinal tract [1], [2], [3]. To regulate expression of genes involved in bile acid synthesis, cholesterol and triglyceride metabolism, FXR binds to DNA as a monomer or a heterodimer with a common partner of nuclear receptors, retinoid X receptor (RXR). FXR agonists include bile acids [e.g. chenodeoxycholic acid (CDCA)] [4] and a synthetic compound GW4064 [5]; FXR antagonists include plant-derived guggulsterone [6] and synthetic AGN34 [7].

In addition to its essential role in regulating lipid metabolism, emerging evidence supports an important role for FXR in intestinal carcinogenesis. Decreased FXR mRNA expression is reported in human colon polyps and even more pronounced in colon adenocarcinomas [8], [9]. Modica et al. showed that by regulating Wnt signaling and apoptosis FXR suppressed intestinal tumorigenesis in both the *Apc^Min+/−^* and chronic colitis mouse models of intestinal neoplasia [10]. Maran et al. showed that FXR-deficient mice had increased intestinal epithelial cell proliferation and tumor development [11]. Smith et al. also demonstrated that the bile salt sodium taurocholate inhibited mouse intestinal adenoma formation through activation of FXR in *Apc^Min+/−^* mice by up-regulating the small heterodimer partner (Shp) and down-regulating cyclin D1 [12]. These data suggest not only that FXR activation enhances apoptosis but also that FXR activation inhibits cell proliferation. However, the molecular mechanisms underlying anti-proliferative actions of FXR remains to be delineated.

Previously, we identified cross-talk between the M3 subtype muscarinic receptor (M3R), a G protein-coupled receptor (GPCR), and EGFR, a receptor tyrosine kinase [13]. We showed that M3R cross-talk with EGFR was mediated by activation of matrix metalloproteinase 7 and release of an EGFR ligand, heparin binding EGF-like growth factor [14]. As a consequence of this interaction, muscarinic agonists stimulate colon cancer cell proliferation [15], [16]. Recently, Giordano et al. showed that FXR inhibited proliferation of MCF-7 breast cancer cell growth by down-regulating expression of HER2, a member of the EGFR family [17]. These results suggested to us that cross-talk between FXR and EGFR might be present in human intestinal epithelial cells. Thus, the objectives of our work were to seek evidence for FXR cross-talk with EGFR and elucidate the ramifications of this interaction. In particular, we asked whether, in human colon epithelial cells, activation and inactivation of FXR results in anti- and pro-proliferative effects, respectively and, what are the molecular mechanisms underlying these actions?

Herein, we report the novel observations that in human colon cancer cells Src kinase mediates cross-talk between FXR and EGFR, thereby controlling cell proliferation. We show that inhibiting FXR activity with guggulsterone stimulates cell proliferation by activating EGFR and its downstream target ERK, whereas stimulating FXR activity with GW4064 inhibits EGFR and ERK phosphorylation and reduces cell proliferation. Notably, using a xenograft model we show that FXR overexpression in human colon cancer cells inhibits tumor growth by attenuating cell proliferation.

**Figure 1:**
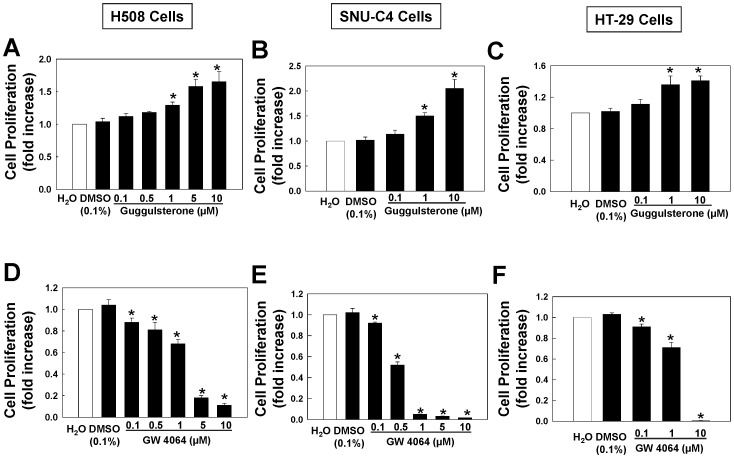
Actions of FXR ligands on proliferation of human colon cancer cells. Dose-response for FXR antagonist guggulsterone-induced proliferation of H508 cells (A), SNU-C4 cells (B) and HT-29 cells (C). Dose-response for FXR agonist GW4064-induced inhibition of proliferation of H508 cells (D), SNU-C4 cells (E) and HT-29 Cells (F). Cells were incubated for 2–5 days at 37°C with FXR ligands at the indicated concentrations. Cell proliferation was measured as described in Materials and Methods. Values represent mean ± SE from at least 3 separate experiments; *p<0.05 vs. control (Student’s *t*-test).

## Materials and Methods

### Ethics Statement

These studies were approved by the Office of Animal Welfare Assurance at the University of Maryland School of Medicine and the Research and Development Committee at the VA Maryland Health Care System (IACUC # 0708012).

**Figure 2:**
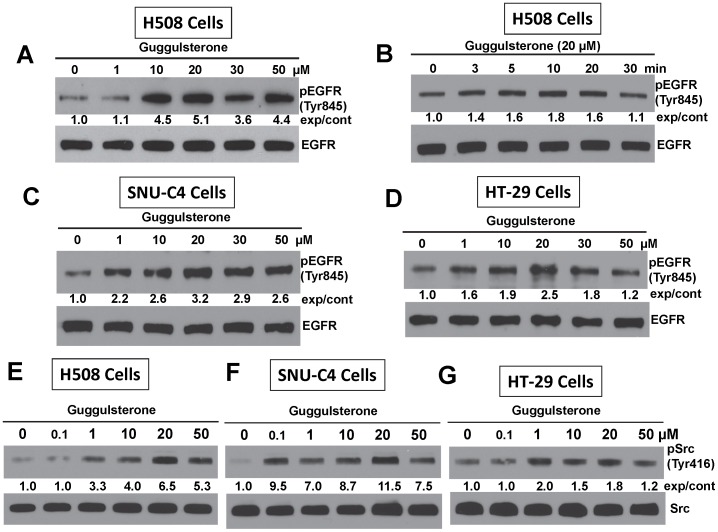
FXR antagonist guggulsterone stimulates phosphorylation of EGFR (Tyr845) and Src (Tyr 416). A. Dose-response for guggulsterone-induced EGFR (Tyr845) phosphorylation in H508 cells. B. Time-course for guggulsterone-induced EGFR (Tyr845) phosphorylation in H508 cells. C. Dose-response for guggulsterone-induced EGFR (Tyr845) phosphorylation in SNU-C4 cells. D. Dose-response for guggulsterone-induced EGFR (Tyr845) phosphorylation in HT-29 cells. Dose-response for guggulsterone-induced Src (Tyr 416) phosphorylation in H508 cells (E), SNU-C4 cells (F) and HT-29 cells (G). For time-course experiments, cells were treated with guggulsterone (20 µM) for the indicated time at 37°C. For dose-response experiments, cells were treated with the indicated concentrations of guggulsterone for 20 min at 37°C. Phosphorylation of EGFR (Tyr845) and Src (Tyr 416) was determined by immunoblotting with anti-phospho-EGFR (Tyr845) and anti-phospho-Src (Tyr 416) antibody, respectively. Immunoblotting for total EGFR or Src was used as a loading control. Numbers between immunoblots represent densitometry. Experimental/control (exp/cont) ratios were calculated after normalizing each test band to the respective EGFR or Src band. Immunoblots are representative of at least 3 separate experiments.

### Materials

Guggulsterone (Z form), GW4064, PP2, PP3, Src inhibitor-1, PD168393, AG1478, PD98059, LY294002 were purchased from Calbiochem. CellTiter 96® AQ_ueous_ One Solution Cell Proliferation Assay (MTS) kit was from Promega; RPMI 1640, DMEM and McCoy’s 5A growth media were from Mediatech.

**Figure 3:**
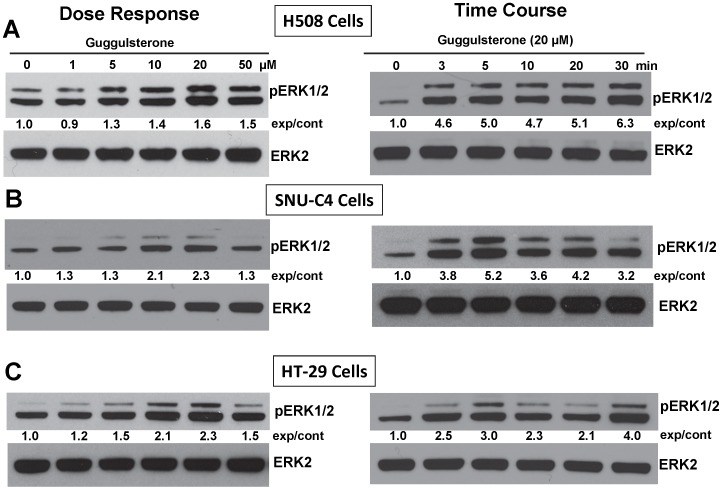
Guggulsterone stimulates phosphorylation of ERK1/2. Dose-response and time-course for guggulsterone-induced phosphorylation of ERK1/2 in H508 (A), SNU-C4 (B) and HT-29 cells (C). For dose-response experiments, cells were treated with the indicated concentrations of guggulsterone for 5 min at 37°C. For time-course experiments, cells were treated with guggulsterone (20 µM) for the indicated times at 37°C. Phosphorylation of ERK1/2 was determined by immunoblotting with anti-phospho-ERK1/2 antibody. Immunoblotting for ERK2 was used as a loading control. Numbers between immunoblots represent densitometry. Experimental/control (exp/cont) ratios were calculated after normalizing each test band to the respective control band. Immunoblots are representative of at least 3 separate experiments.

### Animals

Nude mice (NU/J, stock # 002019) were purchased from the Jackson Laboratory. For all experiments, six-week-old male mice were used. Mice were housed under identical conditions in a pathogen-free room, had free access to commercial rodent chow and water, and were allowed to acclimatize in the vivarium for 2 weeks prior to treatments. Mice were weighed weekly.

**Figure 4:**
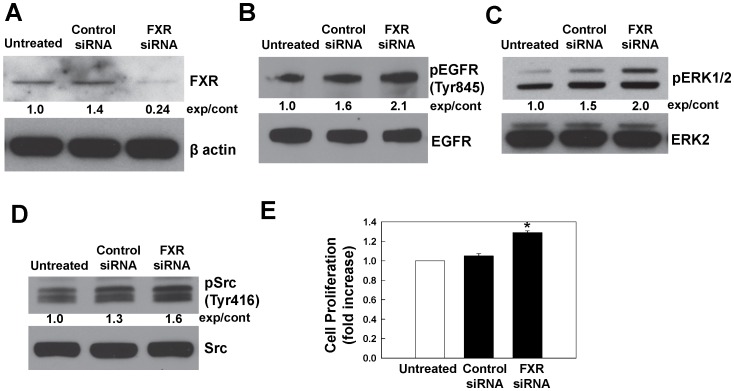
Effects of FXR knockdown on phosphorylation of EGFR, ERK1/2, Src and cell proliferation. A. Immunoblots showing FXR protein levels in untreated, control siRNA-transfected and FXR-siRNA-transfected SNU-C4 cells. SNU-C4 cells in 6-well plates were transfected with either FXR siRNA or control siRNA as described in Materials and Methods. After 48 h, FXR protein levels were determined by immunoblotting. Immunoblotting for β-actin was used as a loading control. Phosphorylation of EGFR (Tyr845), ERK1/2, Src (Tyr416), as shown in panels B, C and D, respectively, was determined by immunoblotting with corresponding antibodies. Immunoblotting for total EGFR, ERK2 or Src was used as a loading control. Numbers between immunoblots represent densitometry. Experimental/control (exp/cont) ratios were calculated after normalizing each test band to the respective control band. E. Untreated, control siRNA-transfected and FXR-siRNA-transfected SNU-C4 cells were incubated for an additional 2 days at 37°C. Cell proliferation was measured as described in Materials and Methods. Values represent mean ± SE from at least 3 separate experiments; *p<0.05 vs. control (Student’s *t*-test).

### Cell Culture

H508 and SNU-C4 human colon cancer cells (ATCC) were grown in RPMI 1640 growth media supplemented with 10% fetal bovine serum. HT-29 human colon cancer cells (ATCC) are maintained in our laboratory in McCoy’s 5A growth media. Adherent cells were passaged weekly at sub-confluence after trypsinization. Cultures were maintained in incubators at 37°C in an atmosphere of 5% CO_2_ and 95% air.

**Figure 5:**
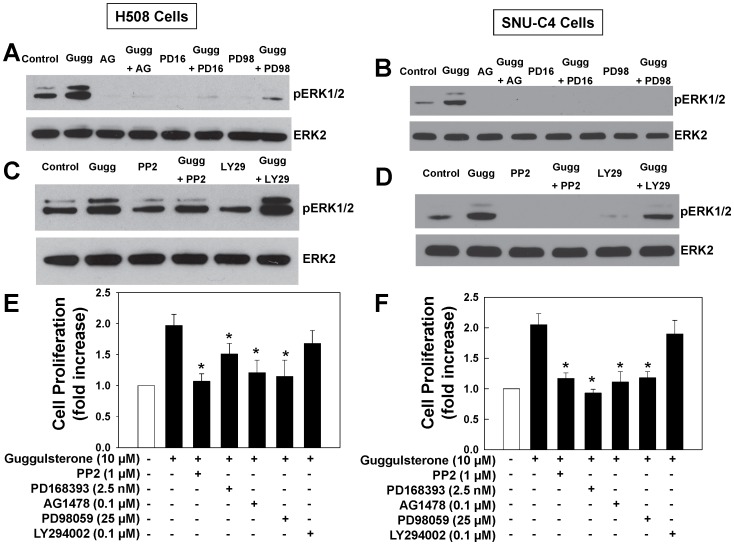
Guggulsterone-induced ERK1/2 phosphorylation and cell proliferation are mediated by Src, EGFR and post-EGFR signaling. H508 cells (A and C) or SNU-C4 cells (B and D) were treated with 20 µM guggulsterone for 20 min at 37°C with or without pre-incubation with EGFR inhibitor AG1478 (AG) and PD168393 (PD16), MEK inhibitor PD98059 (PD98), Src kinase inhibitor PP2 and PI3 kinase inhibitor LY294002 (LY29). ERK1/2 phosphorylation was determined by immunoblotting with anti-phospho-ERK1/2 antibody. Immunoblotting for total ERK2 was used as a loading control. Immunoblots are representative of at least 3 separate experiments. H508 cells (E) or SNU-C4 cells (F) were incubated for 5 days at 37°C with guggulsterone (10 µM) in the presence or absence of Src inhibitor PP2, EGFR inhibitors PD16 and AG, MEK inhibitor PD98 and PI3 kinase inhibitor LY29. Cell proliferation was measured as described in Materials and Methods. Values are means ± SE from at least 3 separate experiments *p<0.05 vs. guggulsterone alone (Student’s *t*-test).

### Cell Proliferation Assay

Cells were seeded in 96-well plates at about 10% confluence and allowed to attach for 24 h. After 18 h serum starvation, 200 µL fresh serum-free growth media containing test agents was added. When chemical inhibitors and antibodies were used, they were added 30 min and 2 h, respectively, before test agents. Cells were incubated for two days for HT29 and SNU-C4 cells or five days for H508 cells at 37°C in an atmosphere of 5% CO_2_ and 95% air without further additions of test agents, and cell proliferation was determined by adding 20 µL CellTiter 96® AQueous One solution (Promega) to each well. After 1- to 2-h incubation at 37°C, absorbance was measured at 490 nm using a 96-well microtiter plate reader (SpectraMax384).

**Figure 6:**
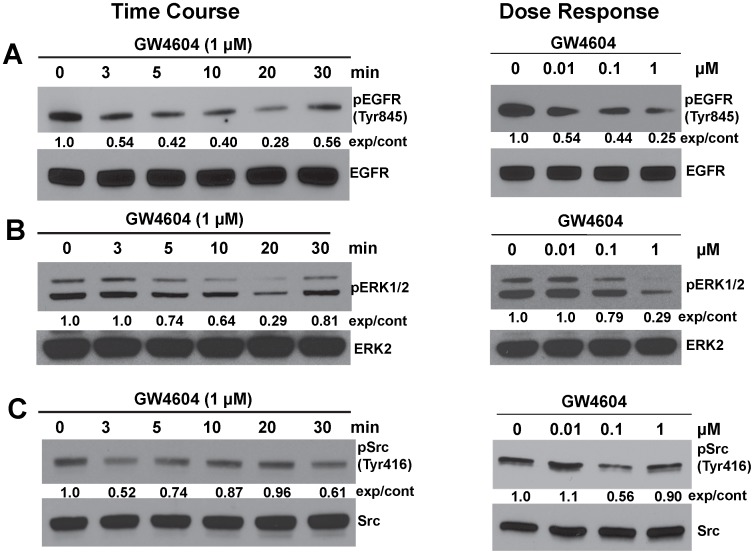
FXR agonist GW4064 inhibits phosphorylation of EGFR (Tyr845), ERK1/2 and Src (Tyr 416) in SNU-C4 cells. A. Time-course and dose-response for GW4064-induced inhibition of EGFR (Tyr845) phosphorylation. B. Time-course and dose-response for GW4064-induced inhibition of ERK1/2 phosphorylation. C. Time-course and dose-response for GW4064-induced inhibition of Src (Tyr 416) phosphorylation. Phosphorylation of EGFR (Tyr845), ERK1/2 and Src (Tyr 416) was determined by immunoblotting with anti-phospho-EGFR (Tyr845), anti-phospho-ERK1/2 and anti-phospho-Src (Tyr 416) antibody, respectively. For time-course experiments, SNU-C4 cells were treated with 1 µM GW4064 for the indicated times at 37°C. For dose-response experiments, cells were treated with the indicated concentrations of GW4064 for 20 min at 37°C. Immunoblotting for total EGFR, ERK2 or Src was used as a loading control. Immunoblots are representative of at least 3 separate experiments. Numbers between immunoblots represent densitometry. Experimental/control ratios were calculated after normalizing each test band to the respective EGFR, ERK2 or Src band.

### Plasmids and Subcloning

Plasmid pCMX-hFXR that contains full-length human FXR and pCMX were generous gifts from Dr. David Mangelsdorf, University of Texas Southwestern Medical Center [18]. Full length hFXR was cut from pCMX-hFXR at Asp718 and BamHI sites and sub-cloned into the pcDNA3.1(+) vector. The resulting plasmid pcDNA3.1hFXR was sequenced in its entirety.

**Figure 7:**
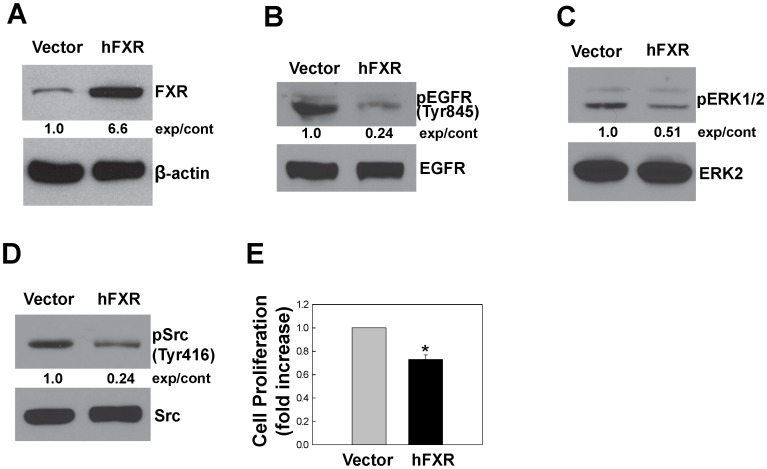
FXR overexpression inhibits phosphorylation of EGFR, ERK and Src, and attenuates cell proliferation. A. Immunoblots showing FXR protein levels in HT-29 cells stably-transfected with empty vector alone or vector containing human FXR (hFXR). Phosphorylation of EGFR (Tyr845), ERK1/2, Src (Tyr416) as shown in panels B, C and D, respectively, was determined by immunoblotting with corresponding antibodies. Immunoblotting for β-actin, total EGFR, ERK2 or Src was used as a loading control. Numbers between immunoblots represent densitometry. Experimental/control (exp/cont) ratios were calculated after normalizing each test band to the respective control band. E. HT-29 cells stably-transfected with empty vector alone or hFXR were incubated for 2 days at 37°C. Cell proliferation was measured as described in Materials and Methods. Values represent mean ± SE from at least 3 separate experiments; *p<0.05 vs. control (Student’s *t*-test).

### Stable Transfection

To generate stably-transfected cells, we first determined the kill-curves for H508, SNU-C4 and HT29 cells with various concentrations of G418. Only HT29 cells produced G418-resistant cells (at 1 mg/mL G418) that overexpressed hFXR, which was determined by immunoblotting with anti-FXR antibody. Vector-transfected and hFXR-transfected cell lines that were derived from multiple G418-resistant clones were used for subsequent *in vivo* studies.

**Figure 8:**
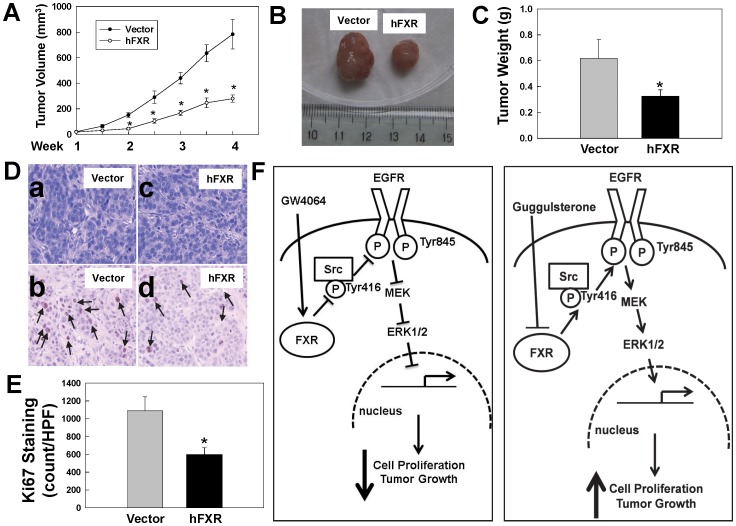
FXR overexpression attenuates growth of HT29 human colon cancer cell xenografts. A. Reduced volume of hFXR-derived tumors compared with vector-derived tumors. B. Representative photos of xenografts from HT-29 cells transfected with vector alone (left) or vector containing hFXR (right). C. Reduced weight of hFXR-derived tumors compared with vector-derived tumors. D. Representative H&E- (upper panels) and Ki67-stained (lower panels) microscopic images of xenografts. *Arrows*, representative Ki67-stained nuclei. E. Reduced Ki67 immunostaining in hFXR-derived tumors compared with vector-derived tumors. Quantification of Ki-67 was performed as described in Materials and Methods. Values represent mean ± SD. *p<0.01 vs. vector-derived xenograft. F. Model depicting proposed mechanisms underlying FXR-mediated, Src-dependent activation of EGFR signaling in colon epithelial cells. Binding of FXR by an agonist (left panel), e.g. GW4064, results in de-phosphorylation of Src (Tyr416). Reduced Src activity results in attenuated EGFR (Tyr485) phosphorylation and downstream ERK1/2 signaling, thereby attenuating cell proliferation. In contrast, binding of FXR by an antagonist (right panel), e.g. guggulsterone, increases phosphorylation of Src (Tyr416). Activated Src phosphorylates EGFR (Tyr845) leading to activated post-EGFR signaling, including activation of ERK1/2, which promotes cell proliferation.

### Antibodies and Immunoblotting

Goat polyclonal and mouse monoclonal anti-FXR antibodies were purchased from Santa Cruz Biotechnology and Sigma-Aldrich, respectively. Rabbit polyclonal anti-Src, rabbit polyclonal anti-phospho-Src (Tyr416), rabbit monoclonal anti-EGFR and rabbit polyclonal anti-phospho-EGFR (Tyr845), rabbit polyclonal anti-ERK1/2 (p44/42) and mouse monoclonal anti-phospho-ERK1/2 were from Cell Signaling Technology, Inc. Neutralizing anti-EGFR antibody (LA1) was from Millipore. Immunoblotting was performed as described previously [13]. For chemical inhibitors (CH223191, PP2, PP3, Src inhibitor-1, AG1478, PD168393, PD98059, U0126, LY294002 and wortmannin) that are soluble only in DMSO, all samples and controls contained the same concentrations of DMSO (0.1% or lower). Densitometry of immunoblots was performed using Adobe Photoshop and normalized to controls.

### Generation of Xenografts

To generate xenografts, 1 million human HT29 colon cancer cells stably transfected with either vector or vector containing the full-length hFXR were injected subcutaneously into the flanks of nude mice in 100 µL RPMI media. A total of six tumors were generated in each group. Tumor size was measured weekly with calipers, and tumor volumes calculated using the formula: tumor volume  = 1/2(length × width^2^). At the end of the study, tumors were excised and weighed.

### FXR Small Interfering RNA Transfection

SNU-C4 cells were seeded in 6- and 96-well plates at approximately 10% confluence and incubated at 37°C for 24 h. Four pooled small interfering RNA (siRNA) duplex oligos targeting human FXR (200 nM; siGENOME SMARTpool; Dharmacon) and control siRNA (200 nM; siGENOME Non-Targeting siRNA Pool#1; Dharmacon) were transfected into H508 cells with DharmaFECT4 transfection agent according to the manufacturer’s instructions. Two days following transfection, cells in the 96-well plates were incubated for an additional five days in serum-free RPMI medium containing TCDD or various agents at 37°C in a CO_2_ incubator; cells in the 6-well plates were harvested for immunoblotting to determine the degree of FXR protein knockdown.

### Immunohistochemistry

Paraffin-embedded sections were stained with mouse monoclonal anti-Ki67 antibody (Bethyl Laboratories) or rabbit anti-cleaved caspase-3 antibody (Cell Signaling Technology) according to manufacturer’s instructions. Quantification of Ki67 staining was performed using Image-Pro Plus software (Media Cybernetics).

### Statistical Analysis

All data are expressed as mean ± SE of at least three independent experiments. Statistical calculations were performed using Student’s un-paired t-test (SigmaPlot, Systat Software, Inc., San Jose, CA). P<0.05 was considered significant.

## Results

### FXR Regulates Proliferation of Human Colon Cancer Cells

To determine whether FXR plays a role in controlling proliferation of human colon cancer cells, we examined the actions of increasing concentrations of a selective FXR antagonist, guggulsterone (Z form), and agonist, GW4064, on proliferation of three commonly-used human cancer cell lines, H508, SNU-C4 and HT-29. For this work, we used GW4064 to activate FXR because chenodeoxycolic acid (CDCA), a natural and specific FXR agonist [4], can also bind and activate muscarinic receptors that are over-expressed in most human colon cancer cell lines {[19], [20]; data not shown}. As shown in Fig. 1A–C, in all three cell lines, treatment with guggulsterone resulted in dose-dependent increases in cell proliferation. In contrast, as shown in Fig. 1D–F, treatment with GW4064 resulted in dose-dependent attenuation of cell proliferation.

### Guggulsterone Stimulates EGFR Phosphorylation

In many human cancers, EGFR plays a central role in promoting cell proliferation [21]. To determine whether guggulsterone-induced cell proliferation was mediated by activation of EGFR, we examined the actions of guggulsterone on EGFR (Tyr845) phosphorylation, which is primarily mediated by Src kinase [22], [23]. As shown in Fig. 2, A and B, in H508 cells guggulsterone induced robust, time- and dose-dependent phosphorylation of EGFR (Tyr845). Using a maximal dose of 20 µM, guggulsterone-induced EGFR (Tyr845) phosphorylation peaked at 10 to 20 min in all three cell lines (data for SNU-C4 and HT-29 cells not shown).

### Guggulsterone Induces Src (Tyr416) Phosphorylation

EGFR (Tyr845) phosphorylation is mediated primarily by Src kinase. Phosphorylation of Src (Tyr416) up-regulates enzyme activity. As shown in Fig. 2E–2G, treatment with guggulsterone stimulated dose-dependent Src (Tyr416) phosphorylation with similar stoichiometry to that observed for EGFR (Tyr845) phosphorylation. Gugglesterone-induced Src (Tyr416) phosphorylation peaked within 20 min in all three colon cancer cell lines (not shown).

### Guggulsterone Induces ERK1/2 Phosphorylation

To determine whether guggulsterone-induced EGFR phosphorylation activates downstream targets of EGFR we examined the actions of guggulsterone on ERK1/2 phosphorylation. As shown in Fig. 3, in all three cell lines guggulsterone induced dose-dependent ERK1/2 phosphorylation with a maximal concentration of 20 µM. Guggulsterone (20 µM)-induced phosphorylation of ERK1/2 peaked within 5 min (Fig. 3).

### Effects of FXR Knockdown with FXR siRNA

To confirm our finding that the FXR antagonist guggulsterone induced phosphorylation of EGFR (Tyr845) and ERK1/2 we examined the effects of FXR knockdown using siRNA in SNU-C4 cells. As shown in Fig. 4A, transfection with FXR siRNA reduced FXR protein levels by more than 80%. As shown in Fig. 4B–D, FXR knockdown increased EGFR (Tyr845), ERK1/2 and Src (Tyr416) phosphorylation. Moreover, as shown in Fig. 4E, FXR knockdown increased cell proliferation. These findings provided confirmatory evidence that the actions of gugglsterone were a consequence of inhibited FXR activity.

### Actions of Chemical Inhibitors on Guggulsterone-induced ERK1/2 Phosphorylation

To further delineate the mechanisms underlying guggulsterone-induced ERK1/2 phosphorylation we examined the actions of inhibitors of EGFR, MEK (the kinase immediately upstream of ERK) and Src kinase. As shown in Fig. 5A and 5B, guggulsterone-induced ERK1/2 phosphorylation was abolished by EGFR inhibitors AG1478 and PD168393, by MEK inhibitor PD98059 and by Src inhibitors PP2 and Src inhibitor-1 (Fig. 5C, 5D and Fig. S1), but not by PP3, an inert PP2 analogue (Fig. S1). In contrast, a PI3 kinase inhibitor LY294002 did not inhibit guggulsterone-induced ERK1/2 activation (Fig. 5C and 5D). These results provided evidence that guggulsterone induced ERK1/2 phosphorylation through Src-mediated activation of EGFR.

### Guggulsterone-induced Cell Proliferation is Mediated by EGFR and Post-EGFR Signaling

To determine whether EGFR and post-EGFR signaling played a key role in mediating guggulsterone-induced cell proliferation, we examined the actions of inhibitors of Src, EGFR and a post-EGFR signaling kinase MEK. As shown in Fig. 5E and 5F, in both H508 and SNU-C4 cells, guggulsterone-induced cell proliferation was nearly abolished by Src inhibitor PP2, EGFR inhibitors PD168393 and AG1478, and MEK inhibitor PD98059. In contrast, the PI3 kinase inhibitor LY294002 had no effect. These results provide evidence that in human colon cancer cells guggulsterone stimulates cell proliferation through Src-mediated activation of EGFR and ERK1/2. However, PI3 kinase does not play a role in either gugglsterone-induced signaling or cell proliferation.

### GW4064 Inhibits Phosphorylation of EGFR, ERK1/2 and Src Kinase

Because inhibition of FXR activity by treatment with guggulsterone or siRNA stimulated cell proliferation that was mediated by Src-dependent cross-talk with EGFR (Figs. 1, 2, 3, 4, 5, Fig. S1), we hypothesized that FXR activation would down-regulate Src, EGFR and ERK1/2 phosphorylation (activity). To test this hypothesis we examined the actions of the FXR agonist GW4064 on phosphorylation of EGFR (Tyr845), ERK1/2 and Src (Tyr 416). Phosphorylation of Src (Tyr416) up-regulates enzyme activity. As shown in Fig. 6, in SNU-C4 cells treatment with GW4064 resulted in time- and dose-dependent reduced phosphorylation of EGFR (Tyr845; Fig. 6A), ERK1/2 (Fig. 6B) and Src (Tyr416; Fig. 6C). These effects were observed within 5 min with as little as 100 nM GW4064 (Fig. 6). These data provide evidence that FXR activation inhibits phosphorylation of EGFR (Tyr845), ERK1/2 and Src (Tyr416), thereby resulting in decreased cell proliferation (Fig. 1D–1F).

### Overexpressing FXR Reduces Cell Proliferation *in vitro*

To determine whether FXR activation inhibits cell proliferation *in vitro*, we examined the effect of FXR overexpression in colon cancer cells. After constructing the plasmid pcDNA3.1hFXR that contained full-length human FXR cDNA (Materials and Methods) we attempted to generate stably-transfected H508 and SNU-C4 cell lines overexpressing FXR in cells but were unsuccessful (data not shown). We speculated that inhibition of proliferation resulting from overexpression of FXR in these cell lines conferred a growth disadvantage for transfected cells. Nonetheless, using HT-29 cells we successfully generated several cell clones overexpressing FXR. As shown in Fig. 7A, one stably-transfected HT-29 cell line overexpressed hFXR by approximately 6.6-fold compared to cells transfected with vector alone. In this cell line FXR overexpression resulted in reduced phosphorylation of EGFR [(Tyr845); Fig. 7B], ERK1/2 (Fig. 7C) and Src [(Tyr416); Fig. 7D], as well as reduced cell proliferation (Fig. 7E).

### Overexpressing FXR Attenuates Tumor Growth in Nude Mice

To investigate the effects of FXR overexpression on *in vivo* cell proliferation we used a mouse xenograft model. Stably-transfected HT-29 cells were used to generate tumor xenografts in nude mice (Materials and Methods). Compared to tumors generated from cells stably transfected with vector alone overexpression of FXR robustly attenuated tumor volumes by 64% (Fig. 8A) and tumor weights by 47% (Fig. 8C). As shown in Fig. 8D and E, Ki67 immunohistochemistry showed that cellular proliferation was reduced 45% in cells overexpressing FXR. In addition, as shown in supplemental Fig. 2, consistent with other reports [10], [11], FXR overexpression resulted in increased colon cancer cell apoptosis. The percentage of apoptotic cells in both vector-derived and hFXR-derived xenografts was less than 1% of total cell number (data not shown). Hence, we conclude that changes in apoptosis contribute little to the larger differences observed in tumor volume and weight in xenografts derived from these cells. Collectively, these results provide evidence that FXR activation decreases cell proliferation thereby resulting in attenuated growth of human colon tumors *in vivo*.

## Discussion

Colorectal cancer (CRC) is the second leading cause of cancer death in the United States. Currently, there is no cure for patients with advanced disease. As in many other cancers, EGFR plays a prominent role in promoting growth of CRC [24], [25]; biological therapies targeting EGFR are FDA-approved for treatment of EGFR-positive advanced CRC [26], [27]. However, cancer cells have developed mechanisms to activate EGFR indirectly through cross-talk with other signaling pathways, which, in turn, may limit the benefits of currently-approved EGFR-targeted biological therapies [28], [29], [30]. For example, in mouse models of CRC and several human colon cancer cell lines including H508 and SNU-C4 cells used in this study, we showed that cholinergic muscarinic receptors cross-talk with EGFR to stimulate cell proliferation, tumor growth and invasion [13], [16], [31], [32], [33], [34].

Findings described in the present study reveal a novel mechanism whereby FXR regulates cell proliferation through EGFR signaling in colon cancer cells. The FXR antagonist guggulsterone robustly stimulated proliferation of three colon epithelial cell lines (Fig. 1), guggulsterone induced time- and dose-dependent increases in EGFR (Tyr845), Src (Tyr416) and ERK1/2 phosphorylation (Figs. 2 and 3) whereas an FXR agonist GW4064 caused time- and dose-dependent decreases in Src (Tyr416), EGFR (Tyr845) and ERK1/2 phosphorylation (Fig. 6). Guggulsterone-induced phosphorylation of EGFR, ERK1/2 and increased cell proliferation were either abolished or strongly attenuated by inhibitors of Src, EGFR and MEK (Fig. 5). The actions of guggulsterone was further validated by transfecting cells with siRNA for FXR, which resulted in increased phosphorylation of EGFR (Tyr845), ERK1/2, Src (Tyr416) and cell proliferation (Fig. 4). In addition, overexpressing FXR in HT-29 cells reduced phosphorylation of EGFR (Tyr845), ERK1/2, Src (Tyr416), and attenuated cell proliferation *in vitro* (Fig. 7) and both cell proliferation and growth of human xenografts in nude mice (Fig. 8). It should be noted that in the absence of treatment with an FXR agonist, the actions of guggulsterone on Src, EGFR and ERK phosphorylation are most likely a consequence of inhibited constitutive FXR activity. Also, although guggulsterone is commonly used experimentally as a selective FXR antagonist, we cannot exclude the possibility that guggulsterone induces phosphorylation of EGFR, Src and ERK by action on a yet unknown protein receptor. Thus, it was important for us to provide confirmatory evidence using an FXR agonist (GW4064), FXR knockdown using specific siRNA and stable FXR overexpression in multiple human cancer cell lines. The resulting observations strongly support our conclusion that in both *in vitro* and *in vivo* human colon epithelial cell models, FXR regulates cell proliferation via Src-mediated cross-talk with EGFR.

As shown in Fig. 8F, based on the present findings we propose a model delineating key steps from FXR-ligand interaction to changes in human colon cancer cell proliferation. FXR activation results in inactivation of Src and EGFR, thereby inhibiting post-EGFR ERK1/2 signaling and attenuating cell proliferation and tumor growth (Fig. 8F, left panel). In contrast, blocking FXR activation increases Src (Tyr416) phosphorylation, which in turn activates EGFR and post-receptor ERK1/2 signaling, thereby resulting in increased cell proliferation and tumor growth (Fig. 8F, right panel).

We showed that FXR modulates cell proliferation through Src-mediated changes in EGFR phosphorylation and post-EGFR signaling, however, the mechanism underlying the interaction between FXR and Src remains to be determined. FXR and Src co-immunoprecipitation experiments did not show co-existence of these two proteins in the same protein complex (data not shown). Hence, we speculate that FXR regulates Src activity indirectly through other intermediaries possibly, including molecules that are present in the cytosolic Src protein complex, e.g., cdc37 and hsp90 [35].

Because guggulsterone exhibits anti-proliferative effects on several malignancies including melanoma, breast carcinoma, prostate cancer and leukemias [36], [37], [38], [39], [40], it was proposed as a potential anti-cancer therapeutic [41]. However, in the present study, we showed that guggulsterone caused robust proliferation of two human colon cancer cell lines through Src-mediated activation of EGFR and post-EGFR signaling. Contradictory observations in different tissues suggest that guggulsterone may have pleotropic effects on cell proliferation that are tissue-specific. These findings warrant caution when considering guggulsterone as a therapeutic agent for cancer.

In the present study, we showed that overexpressing FXR resulted in attenuated cell proliferation and human colon cancer xenograft growth (Fig. 7, 8), findings consistent with the observation that FXR deficiency in mice augmented intestinal epithelial cell proliferation and tumor development [10], [11]. Both uncontrolled cell proliferation and reduced apoptosis are hallmarks of neoplasia. Because activation of FXR increases apoptosis [10], [42] and decreases cell proliferation, and FXR is highly expressed in the intestines [8], targeted FXR overexpression and/or activation may provide an effective modality for treating CRC.

## Supporting Information

Figure S1 **Guggulsterone-induced ERK1/2 phosphorylation is mediated by Src kinase.** A. H508 cells were treated with 20 µM guggulsterone for 20 min at 37°C with or without pre-incubation with Src inhibitor I (SRC1) or PP2 analogue PP3. B. HT-29 cells were treated with 20 µM guggulsterone for 20 min at 37°C with or without pre-incubation with SRC1, PP2 or PP2 analogue PP3. ERK1/2 Phosphorylation was determined by immunoblotting with anti-phospho-ERK1/2 antibody. Immunoblotting for total ERK2 was used as a loading control. Immunoblots are representative of at least 3 separate experiments.
(TIFF)

Figure S2 **Effect of hFXR overexpression on apoptosis in HT-29 xenografts.** A. Representative cleaved caspase-3-stained sections from empty vector-derived tumors or hFXR-derived tumors. Arrows, cleaved caspase-3-stained cells. B. Number of cleaved caspase-3-positive cells in empty vector-derived tumors or hFXR-derived tumors per high power field (200X); bars, SD; n = 6 tumors per group; *p<0.05 vs. vector (Student’s *t*-test).
(TIFF)

## References

[1] Gadaleta RM, van Mil SW, Oldenburg B, Siersema PD, Klomp LW, et al (2010) Bile acids and their nuclear receptor FXR: Relevance for hepatobiliary and gastrointestinal disease. Biochim Biophys Acta 1801: 683–692. 20399894 10.1016/j.bbalip.2010.04.006

[2] Lee FY, Lee H, Hubbert ML, Edwards PA, Zhang Y (2006) FXR, a multipurpose nuclear receptor. Trends Biochem Sci 31: 572–580. 16908160 10.1016/j.tibs.2006.08.002

[3] Wang YD, Chen WD, Moore DD, Huang W (2008) FXR: a metabolic regulator and cell protector. Cell Res 18: 1087–1095. 18825165 10.1038/cr.2008.289

[4] Parks DJ, Blanchard SG, Bledsoe RK, Chandra G, Consler TG, et al (1999) Bile acids: natural ligands for an orphan nuclear receptor. Science 284: 1365–1368. 10334993 10.1126/science.284.5418.1365

[5] Maloney PR, Parks DJ, Haffner CD, Fivush AM, Chandra G, et al (2000) Identification of a chemical tool for the orphan nuclear receptor FXR. J Med Chem 43: 2971–2974. 10956205 10.1021/jm0002127

[6] Urizar NL, Liverman AB, Dodds DT, Silva FV, Ordentlich P, et al (2002) A natural product that lowers cholesterol as an antagonist ligand for FXR. Science 296: 1703–1706. 11988537 10.1126/science.1072891

[7] Dussault I, Beard R, Lin M, Hollister K, Chen J, et al (2003) Identification of gene-selective modulators of the bile acid receptor FXR. J Biol Chem 278: 7027–7033. 12496277 10.1074/jbc.M209863200

[8] De Gottardi A, Touri F, Maurer CA, Perez A, Maurhofer O, et al (2004) The bile acid nuclear receptor FXR and the bile acid binding protein IBABP are differently expressed in colon cancer. Dig Dis Sci 49: 982–989. 15309887 10.1023/b:ddas.0000034558.78747.98

[9] Lax S, Schauer G, Prein K, Kapitan M, Silbert D, et al.. (2011) Expression of the nuclear bile acid receptor/farnesoid X receptor is reduced in human colon carcinoma compared to nonneoplastic mucosa independent from site and may be associated with adverse prognosis. Int J Cancer. 10.1002/ijc.26293 21780109

[10] Modica S, Murzilli S, Salvatore L, Schmidt DR, Moschetta A (2008) Nuclear bile acid receptor FXR protects against intestinal tumorigenesis. Cancer Res 68: 9589–9594. 19047134 10.1158/0008-5472.CAN-08-1791

[11] Maran RR, Thomas A, Roth M, Sheng Z, Esterly N, et al (2009) Farnesoid X receptor deficiency in mice leads to increased intestinal epithelial cell proliferation and tumor development. J Pharmacol Exp Ther 328: 469–477. 18981289 10.1124/jpet.108.145409 PMC2682273

[12] Smith DL, Keshavan P, Avissar U, Ahmed K, Zucker SD (2010) Sodium taurocholate inhibits intestinal adenoma formation in APCMin/+ mice, potentially through activation of the farnesoid X receptor. Carcinogenesis 31: 1100–1109. 20194350 10.1093/carcin/bgq050 PMC2878362

[13] Cheng K, Zimniak P, Raufman JP (2003) Transactivation of the epidermal growth factor receptor mediates cholinergic agonist-induced proliferation of H508 human colon cancer cells. Cancer Res 63: 6744–6750. 14583469

[14] Cheng K, Xie G, Raufman JP (2007) Matrix metalloproteinase-7-catalyzed release of HB-EGF mediates deoxycholyltaurine-induced proliferation of a human colon cancer cell line. Biochem Pharmacol 73: 1001–1012. 17222808 10.1016/j.bcp.2006.11.028 PMC1852457

[15] Cheng K, Raufman JP (2005) Bile acid-induced proliferation of a human colon cancer cell line is mediated by transactivation of epidermal growth factor receptors. Biochem Pharmacol 70: 1035–1047. 16139803 10.1016/j.bcp.2005.07.023

[16] Xie G, Cheng K, Shant J, Raufman JP (2009) Acetylcholine-induced activation of M3 muscarinic receptors stimulates robust matrix metalloproteinase gene expression in human colon cancer cells. Am J Physiol Gastrointest Liver Physiol 296: G755–763. 19221016 10.1152/ajpgi.90519.2008 PMC2670666

[17] Giordano C, Catalano S, Panza S, Vizza D, Barone I, et al (2011) Farnesoid X receptor inhibits tamoxifen-resistant MCF-7 breast cancer cell growth through downregulation of HER2 expression. Oncogene 30: 4129–4140. 21499302 10.1038/onc.2011.124 PMC4482257

[18] Lu TT, Makishima M, Repa JJ, Schoonjans K, Kerr TA, et al (2000) Molecular basis for feedback regulation of bile acid synthesis by nuclear receptors. Mol Cell 6: 507–515. 11030331 10.1016/s1097-2765(00)00050-2

[19] Frucht H, Jensen RT, Dexter D, Yang WL, Xiao Y (1999) Human colon cancer cell proliferation mediated by the M3 muscarinic cholinergic receptor. Clin Cancer Res 5: 2532–2539. 10499630

[20] Yang WL, Frucht H (2000) Cholinergic receptor up-regulates COX-2 expression and prostaglandin E(2) production in colon cancer cells. Carcinogenesis 21: 1789–1793. 11023534 10.1093/carcin/21.10.1789

[21] Yarden Y (2001) The EGFR family and its ligands in human cancer. signalling mechanisms and therapeutic opportunities. Eur J Cancer 37 Suppl 4S3–8. 10.1016/s0959-8049(01)00230-1 11597398

[22] Biscardi JS, Maa MC, Tice DA, Cox ME, Leu TH, et al (1999) c-Src-mediated phosphorylation of the epidermal growth factor receptor on Tyr845 and Tyr1101 is associated with modulation of receptor function. J Biol Chem 274: 8335–8343. 10075741 10.1074/jbc.274.12.8335

[23] Sato K, Sato A, Aoto M, Fukami Y (1995) c-Src phosphorylates epidermal growth factor receptor on tyrosine 845. Biochem Biophys Res Commun 215: 1078–1087. 7488034 10.1006/bbrc.1995.2574

[24] McKay JA, Murray LJ, Curran S, Ross VG, Clark C, et al (2002) Evaluation of the epidermal growth factor receptor (EGFR) in colorectal tumours and lymph node metastases. Eur J Cancer 38: 2258–2264. 12441262 10.1016/s0959-8049(02)00234-4

[25] Wan CW, McKnight MK, Brattain DE, Brattain MG, Yeoman LC (1988) Different epidermal growth factor growth responses and receptor levels in human colon carcinoma cell lines. Cancer Lett 43: 139–143. 3264518 10.1016/0304-3835(88)90226-1

[26] Overman MJ, Hoff PM (2007) EGFR-targeted therapies in colorectal cancer. Dis Colon Rectum 50: 1259–1270. 17566832 10.1007/s10350-007-0228-3

[27] Venook AP (2005) Epidermal growth factor receptor-targeted treatment for advanced colorectal carcinoma. Cancer 103: 2435–2446. 15880563 10.1002/cncr.21123

[28] Bhola NE, Grandis JR (2008) Crosstalk between G-protein-coupled receptors and epidermal growth factor receptor in cancer. Front Biosci 13: 1857–1865. 17981673 10.2741/2805

[29] Hu T, Li C (2010) Convergence between Wnt-beta-catenin and EGFR signaling in cancer. Mol Cancer 9: 236. 20828404 10.1186/1476-4598-9-236 PMC2944186

[30] van der Veeken J, Oliveira S, Schiffelers RM, Storm G, van Bergen En Henegouwen PM, et al (2009) Crosstalk between epidermal growth factor receptor- and insulin-like growth factor-1 receptor signaling: implications for cancer therapy. Curr Cancer Drug Targets 9: 748–760. 19754359 10.2174/156800909789271495

[31] Belo A, Cheng K, Chahdi A, Shant J, Xie G, et al (2011) Muscarinic receptor agonists stimulate human colon cancer cell migration and invasion. Am J Physiol Gastrointest Liver Physiol 300: G749–G760. 21273532 10.1152/ajpgi.00306.2010 PMC3094147

[32] Raufman JP, Cheng K, Saxena N, Chahdi A, Belo A, et al (2011) Muscarinic receptor agonists stimulate matrix metalloproteinase 1-dependent invasion of human colon cancer cells. Biochem Biophys Res Commun 415: 319–324. 22027145 10.1016/j.bbrc.2011.10.052 PMC3221914

[33] Belo A, Cheng K, Chahdi A, Shant J, Xie G, et al (2011) Muscarinic receptor agonists stimulate human colon cancer cell migration and invasion. Am J Physiol Gastrointest Liver Physiol 300: G749–760. 21273532 10.1152/ajpgi.00306.2010 PMC3094147

[34] Raufman JP, Samimi R, Shah N, Khurana S, Shant J, et al (2008) Genetic ablation of M3 muscarinic receptors attenuates murine colon epithelial cell proliferation and neoplasia. Cancer Res 68: 3573–3578. 18483237 10.1158/0008-5472.CAN-07-6810 PMC2577901

[35] Haarmann-Stemmann T, Bothe H, Abel J (2009) Growth factors, cytokines and their receptors as downstream targets of arylhydrocarbon receptor (AhR) signaling pathways. Biochem Pharmacol 77: 508–520. 18848820 10.1016/j.bcp.2008.09.013

[36] Ahn KS, Sethi G, Sung B, Goel A, Ralhan R, et al (2008) Guggulsterone, a farnesoid X receptor antagonist, inhibits constitutive and inducible STAT3 activation through induction of a protein tyrosine phosphatase SHP-1. Cancer Res 68: 4406–4415. 18519703 10.1158/0008-5472.CAN-07-6696

[37] An MJ, Cheon JH, Kim SW, Kim ES, Kim TI, et al (2009) Guggulsterone induces apoptosis in colon cancer cells and inhibits tumor growth in murine colorectal cancer xenografts. Cancer Lett 279: 93–100. 19232820 10.1016/j.canlet.2009.01.026

[38] Sarfaraz S, Siddiqui IA, Syed DN, Afaq F, Mukhtar H (2008) Guggulsterone modulates MAPK and NF-kappaB pathways and inhibits skin tumorigenesis in SENCAR mice. Carcinogenesis 29: 2011–2018. 18684729 10.1093/carcin/bgn180 PMC2722858

[39] Shishodia S, Aggarwal BB (2004) Guggulsterone inhibits NF-kappaB and IkappaBalpha kinase activation, suppresses expression of anti-apoptotic gene products, and enhances apoptosis. J Biol Chem 279: 47148–47158. 15322087 10.1074/jbc.M408093200

[40] Singh SV, Choi S, Zeng Y, Hahm ER, Xiao D (2007) Guggulsterone-induced apoptosis in human prostate cancer cells is caused by reactive oxygen intermediate dependent activation of c-Jun NH2-terminal kinase. Cancer Res 67: 7439–7449. 17671214 10.1158/0008-5472.CAN-07-0120

[41] Kapoor S (2008) Guggulsterone: a potent farnesoid X receptor antagonist and its rapidly evolving role as a systemic anticarcinogenic agent. Hepatology 48: 2090–2091. 10.1002/hep.22601 18980177

[42] Swales KE, Korbonits M, Carpenter R, Walsh DT, Warner TD, et al (2006) The farnesoid X receptor is expressed in breast cancer and regulates apoptosis and aromatase expression. Cancer Res 66: 10120–10126. 17047076 10.1158/0008-5472.CAN-06-2399

