# Supplementary figures and images for: Src-Mediated Cross-Talk between Farnesoid X and Epidermal Growth Factor Receptors Inhibits Human Intestinal Cell Proliferation and Tumorigenesis
Source: PLoS One. 2012 Oct 31;7(10):e48461. doi: 10.1371/journal.pone.0048461 (PMC3485230; doi:10.1371/journal.pone.0048461)

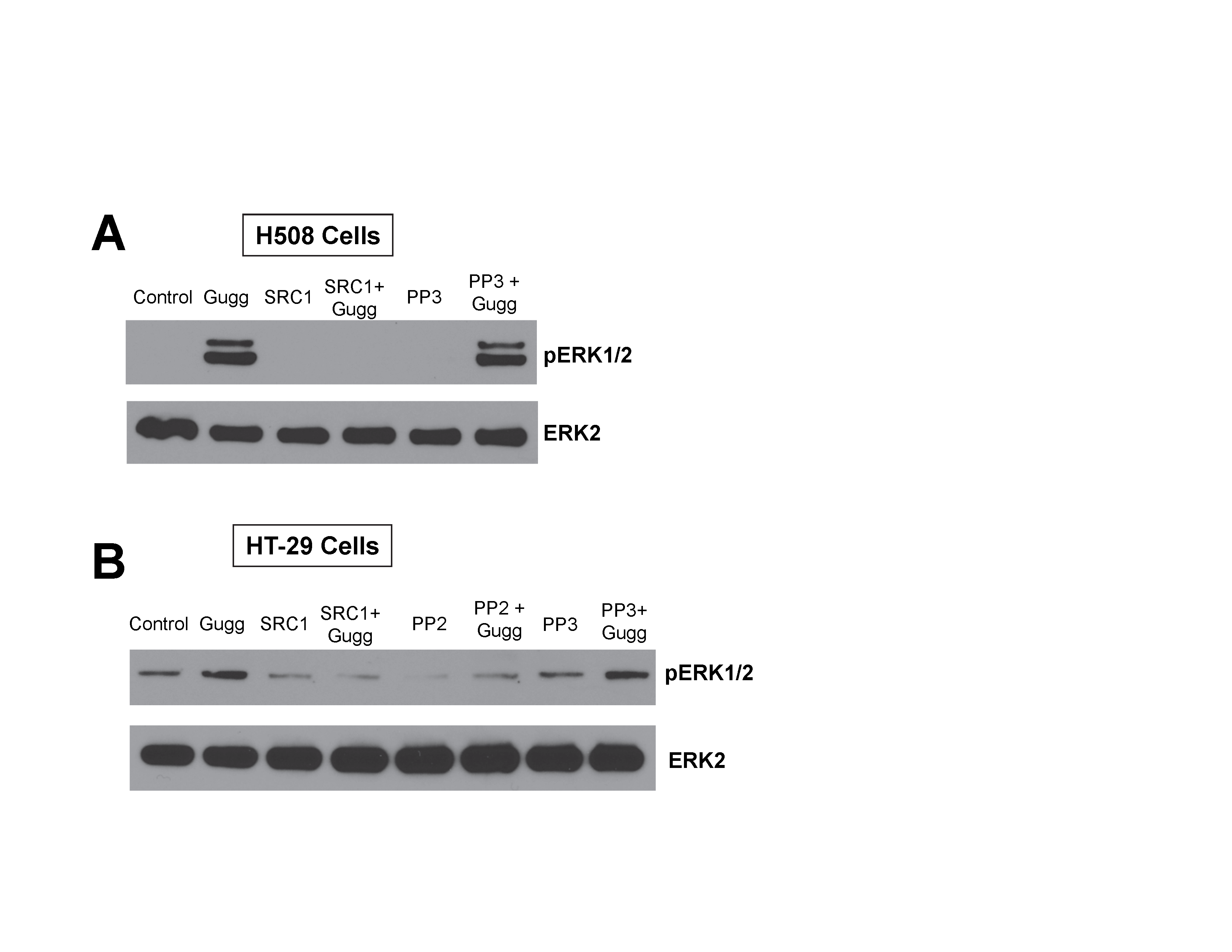

Supplement: Figure S1 — Guggulsterone-induced ERK1/2 phosphorylation is mediated by Src kinase. A. H508 cells were treated with 20 µM guggulsterone for 20 min at 37°C with or without pre-incubation with Src inhibitor I (SRC1) or PP2 analogue PP3. B. HT-29 cells were treated with 20 µM guggulsterone for 20 min at 37°C with or without pre-incubation with SRC1, PP2 or PP2 analogue PP3. ERK1/2 Phosphorylation was determined by immunoblotting with anti-phospho-ERK1/2 antibody. Immunoblotting for total ERK2 was used as a loading control. Immunoblots are representative of at least 3 separate experiments. (TIFF) [file pone.0048461.s001.tiff]

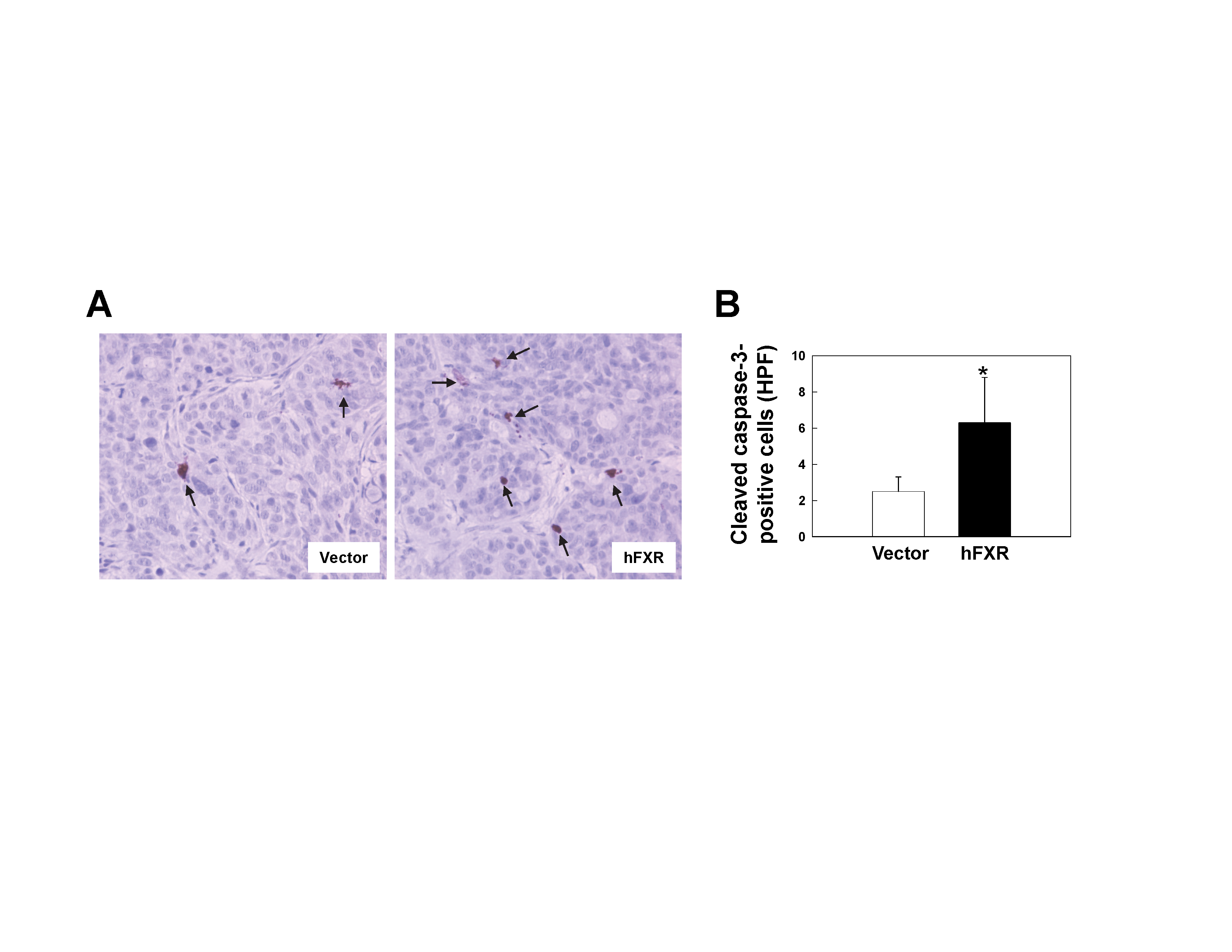

Supplement: Figure S2 — Effect of hFXR overexpression on apoptosis in HT-29 xenografts. A. Representative cleaved caspase-3-stained sections from empty vector-derived tumors or hFXR-derived tumors. Arrows, cleaved caspase-3-stained cells. B. Number of cleaved caspase-3-positive cells in empty vector-derived tumors or hFXR-derived tumors per high power field (200X); bars, SD; n = 6 tumors per group; *p<0.05 vs. vector (Student’s t-test). (TIFF) [file pone.0048461.s002.tiff]
